# First-Trimester Sequential Screening for Preeclampsia Using Angiogenic Factors: Study Protocol for a Prospective, Multicenter, Real Clinical Setting Study

**DOI:** 10.3389/fcvm.2022.931943

**Published:** 2022-07-26

**Authors:** Cristina Trilla, Cristina Luna, Silvia De León Socorro, Leire Rodriguez, Aina Ruiz-Romero, Josefina Mora Brugués, Taysa Benítez Delgado, Marta Fabre, Alicia Martin Martínez, Sara Ruiz-Martinez, Elisa Llurba, Daniel Oros

**Affiliations:** ^1^Obstetrics and Gynecology Department, Hospital de la Santa Creu i Sant Pau, Universitat Autònoma de Barcelona, Barcelona, Spain; ^2^Red RICORS “Primary Care Interventions to Prevent Maternal and Child Chronic Diseases of Perinatal and Developmental Origin”, RD21/0012/0001, Instituto de Salud Carlos III, Madrid, Spain; ^3^Institut d'Investigació Biomèdica Sant Pau–IIB Sant Pau, Barcelona, Spain; ^4^Obstetrics Department, Aragon Institute of Health Research (IIS Aragon), Hospital Clínico Universitario Lozano Blesa, Zaragoza, Spain; ^5^Department of Obstetrics and Gynecology, Complejo Hospitalario Universitario Insular, Materno Infantil, Las Palmas, Spain; ^6^Department of Obstetrics and Gynecology, Biocruces Bizkaia Health Research Institute, Osakidetza, University of the Basque Country, Cruces University Hospital, Bilbao, Spain; ^7^Department of Obstetrics and Gynaecology, Hospital Universitari Son Llàtzer, Palma de Mallorca, Spain; ^8^Biochemistry Department, Hospital de la Santa Creu i Sant Pau, Universitat Autònoma de Barcelona, Barcelona, Spain; ^9^Biochemistry Department, Complejo Hospitalario Universitario Insular, Materno Infantil, Las Palmas, Spain; ^10^Biochemistry Department, Aragon Institute of Health Research (IIS Aragon), Hospital Clínico Universitario Lozano Blesa, Zaragoza, Spain

**Keywords:** screening, first trimester, sequential, preeclampsia, growth restriction

## Abstract

**Introduction:**

The incidence of preeclampsia (PE) is about 2–8%, making it one of the leading causes of perinatal morbidity and maternal mortality in the world. Early prophylactic low dose administration (150 mg) of acetylsalicylic acid is associated with a significant reduction in the incidence of early-onset PE, intrauterine growth restriction (IUGR), and neonatal mean stay in the intensive care unit (ICU). Universal implementation of a first-trimester screening system including angiogenic and antiangiogenic markers [the Placental Growth Factor (PlGF) and/or soluble fms-like Tyrosine Kinase-1 (sFlt-1)] has shown a prediction rate of 90% for early-onset PE but entails a high financial cost. The aim of this study is to determine the predictive and preventive capacity of a universal PE first-trimester two-step sequential screening model, determining the PlGF only in patients previously classified as intermediate risk by means of a multivariate model based on resources already used in the standard pregnancy control, in a real clinical setting. We hypothesize that this screening model will achieve similar diagnostic performance as the universal determination of PlGF but at a lower economic cost.

**Methods and Analysis:**

This is a prospective, multicentric, cohort study in a real-world clinical setting. Every singleton pregnancy will be recruited at the routine first pregnancy visit. In a first step, the first-trimester risk of PE will be calculated using a multivariate Gaussian distribution model, based on medical history, mean blood pressure, Pregnancy-Associated Plasma Protein A (PAPP-A), and Uterine Artery Doppler Pulsatility Index (UTPI). Patients will be classified into three risk groups for PE: (1) risk ≥ 1/50, high-risk with no further testing (blinded PlGF); (2) risk between 1/51 and 1/500, medium-risk requiring further testing; and (3) risk ≤ 1/501, low-risk with no further testing. In a second step, the PlGF will only be determined in those patients classified as intermediate risk after this first step, and then reclassified into high- or low-risk groups. Prophylactic administration of aspirin (150 mg/day) will be prescribed only in high risk patients. As a secondary objective, sFlt-1 values will be blindly determined in patients with high and intermediate risk to assess its potential performance in the screening for PE.

**Ethics and Dissemination:**

The study will be conducted in accordance with the principles of Good Clinical Practice. This study is approved by the Aragon Research Ethics Committee (CEICA) on 3 July 2020 (15/2020).

**Clinical Trial Registration:**

ClinicalTrials.gov, identifier: NCT04767438.

## Introduction

The incidence of preeclampsia (PE) is about 2–8%, making it one of the leading causes of perinatal morbidity, and responsible for 10–15% of the maternal deaths in the world (1, 2). Women with PE present a higher risk of cardiovascular morbidity, multisystemic complications, and a long-term increased risk for cardiovascular diseases (3) and Mortality (4). Early-onset PE (diagnosed before 32 weeks of gestation) is also frequently associated with intrauterine growth restriction (IUGR), and therefore affecting perinatal outcomes and long-term offspring development (5, 6). Late-onset PE, perhaps clinically less severe but much more common, is also an important cause of maternal and neonatal morbidity and mortality with long-term consequences (7).

Although a complete understanding of the pathogenesis of PE remains unclear, the current theory suggests a two-stage process. The first stage is caused by shallow invasion of the trophoblast, resulting in inadequate remodeling of the spiral arteries. This is presumed to lead to the second stage, which involves the maternal response to endothelial dysfunction and imbalance between angiogenic and antiangiogenic factors (8). In PE and IUGR, this vascular remodeling does not exist or it is incomplete, leading to placental hypoperfusion associated with endothelial cell dysfunction (9). For a correct placental development, a balance between the production of angiogenic (Placental Growth Factor, PlGF) and antiangiogenic factors (soluble Fms-like tyrosine kinase-1, sFlt-1) is required. PIGF and sFlt-1 are partially produced by the syncytiotrophoblast. Angiogenic biomarkers (PlGF and sFlt-1) have shown their ability to predict PE and its complications (10).

We have effective preventive strategies to reduce the incidence of PE. The nightly low dose (150 mg) administration of acetyl salicylic acid (ASA) before 16 weeks of gestational age until term is associated with a 90% reduction in the incidence of preterm-PE (11, 12) and IUGR (13, 14), as well as with a 70% reduction of the neonatal mean stay in intensive care units (ICUs) (15). Therefore, it is essential to develop universal PE screening strategies during the first trimester of pregnancy to determine the risk of each patient, selecting those high-risk candidates to start as soon as possible for the prophylactic treatment with low-dose ASA (16, 17).

Universal PE screening has been proven to be cost-effective (18). PE screening models based on maternal demographic characteristics and risk factors have been proposed (19), with a very low detection rate and an excess in false-positive rate (20). Hence, different multivariate models have been developed. A competitive risk approach at 11–13 weeks by means of a combination of maternal characteristics (age, parity, medical history as thrombophilia, nephrological diseases, and chronic hypertension), maternal biophysical variables [mean arterial pressure (MAP) and Doppler of Uterine Arteries (Uterine Artery Pulsatility Index, UTPI)], and biochemical variables (PlGF) has shown a prediction rate of 90% for early-PE and 75% for late-onset PE, with a 10% false-positive rate (21, 22). Additionally, observational retrospective studies have reported that including antiangiogenic biomarkers such as sFlt-1 could improve the first-trimester screening performance (23).

Unfortunately, universal implementation of any first-trimester screening system, such as angiogenic and antiangiogenic markers (PlGF and/or sFlt-1), entails a high financial cost, unaffordable for many healthcare systems. For that reason, more economical screening alternatives have been explored. In this line, taking advantage of the resources used on a regular basis for the screening of chromosomal abnormalities, an alternative multivariate model based on maternal characteristics, MAP, and Uterine Arteries Pulsatility Index combined with Pregnancy-Associated Plasma Protein A levels (PAPP-A), has shown a detection rate of early-onset PE of 80.8% and for late-onset PE of 39.6%, with a 10% false-positive rate (24).

Recent retrospective studies with large sample sizes (25, 26) suggest that a two-step screening protocol, performing only angiogenic markers in 30% of the population classified as moderate or high risk of PE after an economical first step (24), would correctly select high-risk patients for developing PE, but lower-economic cost. However, these results come from retrospective cohorts after universal screening models with PlGF, and therefore with potential intervention biases.

The aim of this study is to prospectively determine, in a real clinical setting, the predictive and preventive capacity of a universal PE first trimester two-step sequential screening model, determining PlGF only in those patients previously classified as intermediate risk by means of a multivariate model based on medical history, MAP, PAPP-A, and UTPI. We hypothesize that this screening model will achieve similar diagnostic performance as the universal determination of PlGF but at a lower economic cost.

As a secondary objective, in every intermediate and high-risk patient, sFlT-1 values will be determined to assess their potential performance in the screening for PE. Even though this data will be blinded to the researchers and will not be considered for the clinical management. We also evaluate the predictive and preventive capacity of this first-trimester sequential screening model for the development of IUGR.

## Methods And Analysis

### Study Design

This is a multicentric prospective cohort study in a real clinical setting. The study will be conducted in five tertiary Spanish hospitals: Hospital de la Santa Creu i Sant Pau (Barcelona), Complejo Hospitalario Universitario Insular Materno Infantil (Las Palmas), Hospital Universitario de Cruces (Bilbao), Hospital Son Llàtzer (Mallorca), and Hospital Clínico Universitario Lozano Blesa (Zaragoza).

### Study Population and Groups

Participants will be recruited at the routine first pregnancy visit with an obstetrics specialist, always before the 14 weeks of gestation, based on the eligibility criteria presented in Table 1, and followed until delivery. Written informed consent will be obtained from all recruited patients. This study is approved by the Aragon Research Ethics Committee (CEICA) on 3 July 2020 (15/2020). Obstetricians will present the study to all the eligible patients, explaining the study, offering participation, and requesting written informed consent.

**Table 1:** Inclusion and exclusion criteria.

**Inclusion criteria**	**Exclusion criteria**
• Singleton pregnancies • Gestational age between 8 and 13+6 weeks according to first trimester crown-rump length • Could receive regular follow-up • Written informed consent	• Abnormal karyotype, structural abnormalities or congenital infections at inclusion • Multiple pregnancies

### Intervention

We consider the first-trimester universal screening for PE as a routine clinical practice. Therefore, the first-trimester risk of PE will be calculated in every patient using a multivariate Gaussian distribution model validated in our population, based on maternal conditions, biophysical markers, and maternal serum PAPP-A (taken routinely for trisomy screening) (Table 2). No angiogenic factors will be used to determine the risk of PE at this first step.

**Table 2:** Variables included in the first-step multivariate model for the estimation of the risk of preeclampsia (PE).

Maternal factors
• Age
• Ethnicity
• Weight
• Height
• Smoking status
• Parity
• History of preeclampsia
• Pre-existing diabetes
• Pre-existing hypertension
• Thrombophilia
• Renal diseases
• Autoimmune conditions
Biophysical markers
• Mean arterial pressure (MAP)
• Mean uterine arteries pulsatility index
Blood test samples
• Pregnancy-associated plasma protein A (PAPP-A) (MoMs)

**Figure 1:**
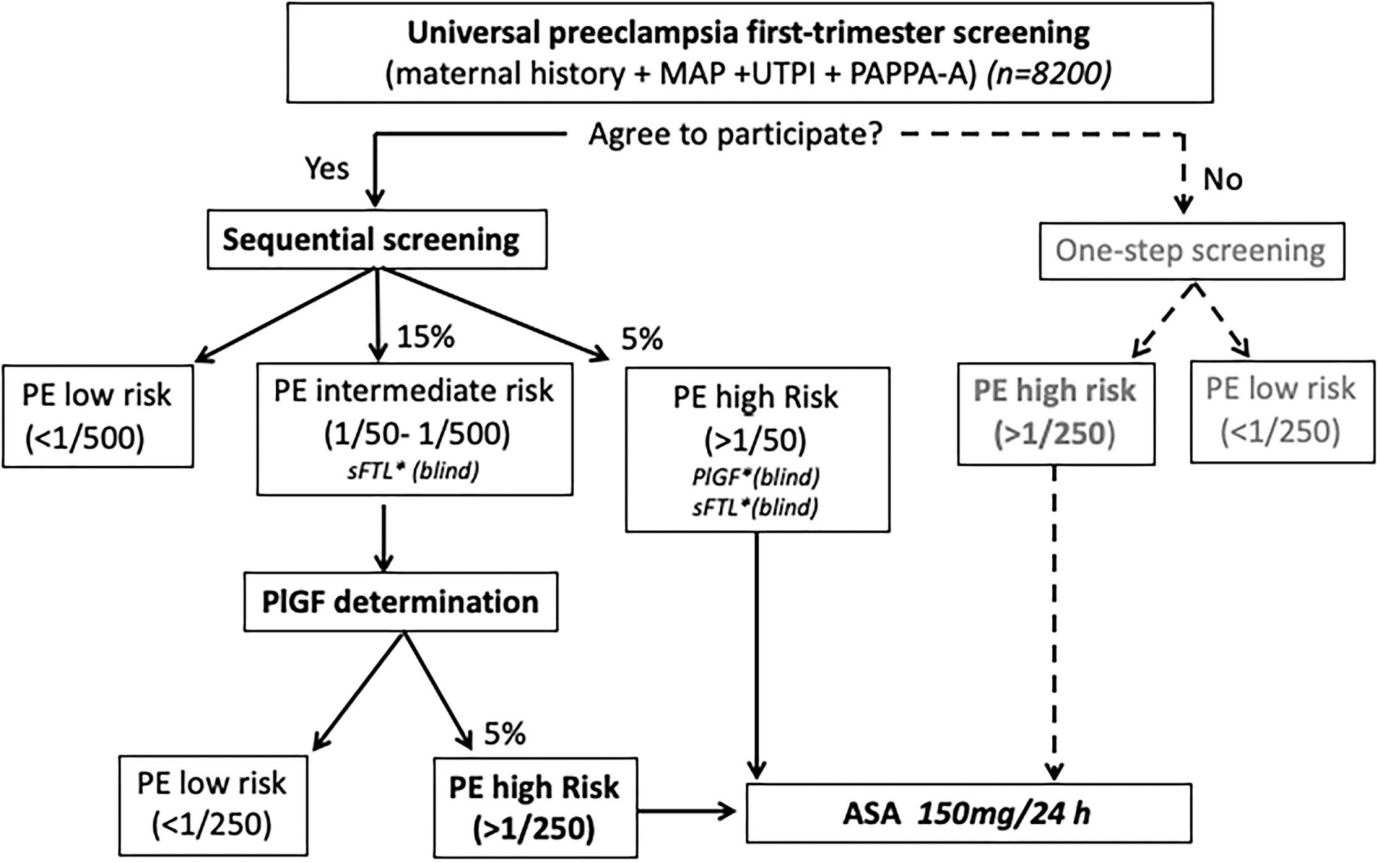
Study protocol algorithm.

All patients who meet the inclusion criteria will be offered the opportunity to participate in the study. If they do not wish to participate, in accordance with a previous pilot study, a risk cut-off of ≥1:250 for early PE will be considered high risk, and therefore prophylactic treatment with ASA 150 mg will be recommended (Figure 1).

In those patients who agree to participate in the study, a two-step contingent sequential screening model will be applied (Figure 1). According to the estimated risk of PE in the first screening step, three risk groups of early PE will be defined: (1) risk ≥ 1/50, high-risk with no further testing (blinded PlGF), (2) risk between 1/51 and 1/500, medium-risk requiring further testing, and (3) risk ≤ 1/501, low-risk with no further testing. According to a previous pilot study performed in the Hospital de la Santa Creu i Sant Pau and Hospital Universitario de Cruces, we expect to find 10% of high-risk patients and 20% of intermediate-risk after the first step of the screening. These results agree with those published by other authors (25).

For the second stage, serum concentrations of PlGF will be determined in the medium-risk group, giving a final risk at a cut-off value of 1/250. Prophylactic treatment with ASA (150 mg) up to 36 weeks of gestation will be recommended in patients classified as high risk of PE either in the first or second step of the model.

With the intention of performing secondary analysis at the end of the study but never to be considered for clinical decisions, the levels of maternal serum sFlT-1 will be determined and blinded for clinicians in patients with an early PE risk ≥ 1/500 after the first step of screening (Figure 1).

In Spain, all pregnancies undergo routine ultrasound scans at ~12, 20, and 36 weeks of gestation. All women with a high risk for preterm PE will be scheduled for an additional scan at approximately 28 weeks.

## Measurements And Outcomes

The most important aim of this project is to develop an efficient first-trimester screening of PE using resources already used during pregnancy control. All the clinical data, sonography, and blood test results will be collected during the regular pregnancy control without the need for additional appointments, explorations, or extraordinary blood extractions.

Maternal characteristics will be prospectively recorded at the time of recruitment. Pregnancy outcomes will be registered during pregnancy and confirmed after delivery. Fetal crown-rump length (CRL) (27), and transabdominally uterine artery Doppler will be determined in all patients at the routine first-trimester ultrasound (11^0^-13^6^ weeks) by experienced fetal medicine specialists. Blood pressure (BP) will be measured once, after 5-min of rest with women seated at the time of inclusion. The MAP will be calculated as: diastolic BP + (systolic–diastolic BP)/3.

First-trimester routine blood samples will be performed between 10^0^ and 13^6^ weeks. PAPP-A (taken routinely for trisomy screening) will be initially determined in every patient. Serum remaining samples will be stored at −80°C, to be able to analyze PlGF and sFlT-1 after the first-trimester ultrasound in those patients with an estimated risk of PE risk ≥ 1/500 after the first step of screening. The serum will be separated by centrifugation at 1,500 g for 10 minutes at 4°C, and concentrations of PAPP-A, PlGF, and sFlT-1 will be determined by electrochemiluminescence immunoassays, fully automated on the Cobas e 601 analyzer, Roche Diagnostics. Multiples of the median (MoM) values for PAPP-A, PlGF, and sFlT-1, calculated from locally derived normal medians using the above-mentioned multivariate Gaussian distribution model, will be considered for analysis. To calculate the risk of PE, we will use the SsdwLab6 version 6.1 package (SBP Soft 2007 S.L.), previously developed in the pilot study.

The main outcome is the diagnosis of PE during pregnancy, according to the definition of the International Society for the Study of Hypertension in Pregnancy (ISSHP). Thus, the diagnosis will be based on systolic BP ≥ 140 mmHg or diastolic BP ≥ 90 mmHg on repeated occasions after 20 weeks' gestation, and proteinuria [dipstick urinalysis ≥ 1+ or protein/creatinine ratio ≥ 30 mg/mmol (0.3 mg/mg)] or another maternal organ dysfunction. PE will be classified according to gestational age at delivery into early-onset (<34 weeks), preterm (<37 weeks), and term (>37 weeks). Table 3 describes the secondary outcomes that will be assessed during the project.

**Table 3:** Study outcomes.

Primary outcomes
• Preeclampsia diagnoses during pregnancy (International Society for the Study of Hypertension in Pregnancy—ISSHP) (28)
Secondary outcomes
• Early-onset Preeclampsia: diagnosed before 32 weeks (28)
• Severe preeclampsia (ISSHP) (28)
• Pregnancy-induced hypertension (28)
• Birth weight below the 10th percentile (29)
• Intrauterine Growth Restriction (30)
• Perinatal mortality (>22 weeks of pregnancy - <28 days postpartum).
• Neonatal acidosis (arterial pH <7.10 + base excess >12 mEq/L)
• Neonatal Intensive Care Unit admission (days)
• Significant neonatal morbidity [convulsions, intraventricular hemorrhage >III grade, periventricular leukomalacia, hypoxic-ischemic encephalopathy, abnormal electroencephalogram, necrotizing enterocolitis, acute renal failure (serum creatinine >1.5 mg/dL) or heart failure (requiring inotropic agents)].
• Gestational age at birth
• Type of delivery (vaginal, instrumental, cesarean section)
• Economic cost the screening (euros)

### Data Management

The processing of the data will be carried out in accordance with the current legislation, specifically article 28 of Regulation (EU) 2016/679 of the European Parliament and of the Council of 27 April 2016 regarding the protection of natural persons regarding the processing of personal data and the free circulation of these data, and in accordance with the provisions of Organic Law 3/2018, of 5 December, on the Protection of Personal Data and guarantee of digital rights. The patient database will be anonymous and codified. Data checks will be regularly performed to ensure data quality. The number of eligible, included, and excluded of patients will be recorded. Withdrawals will also record as detailed as possible.

## Data Analysis

### Sample Size

According to the previously described inclusion criteria and considering the number of pregnancies controlled and delivered among all participating centers during the study period, we estimate that 8,200 patients will be potentially invited to participate in the study.

As the incidence of PE in Spain is ~3% of pregnancies, approximately 246 patients of our sample will develop PE. We could assume that at least 80% of the patients will consent to participate in the study, meaning a final sample of 6,560 singleton pregnancies. Based on a pilot study previously performed, we estimate that 30% of the patients (which means approximately 1,968 pregnancies) will be classified as intermediate or high risk after the first-trimester screening for PE. This sample size assures enough statistic power, external validity, and extrapolation of the results to the clinical practice.

### Statistical Analysis

Continuous variables will be expressed as means ± standard deviation (SD) or median [interquartile range (IQR)], where appropriate. Categorical variables will be expressed as counts (percentages). The variables in the study will be presented using descriptive statistics. Associations between variables were evaluated using Student's *t*-test, chi-square test, or Mann–Whitney's *U*-test where appropriate. A multivariate analysis will be performed using the binomial logistic regression. The diagnostic performance of the contingent screening model will be evaluated by determining the sensitivity, specificity, screen-positive rate, positive predictive value (PPV), and negative predictive value (NPV) for early PE. All reported *p*-values are 2-sided and unequal variances were assumed. A *p* < 0.05 will be used to define statistical significance. Statistical analyses will be performed with the IBMSPSS software program, v.26.0 (IBM-SPSS Inc., Chicago, IL; USA).

## Discussion

Since we have an effective preventive treatment with early administration of low dose ASA, universal screening for PE is cost effective (18), and a clinical and moral obligation. The objective of this project is to validate a first-trimester screening of PE protocol that guarantees access to all pregnant women, without sacrificing quality. For this reason, in the first step of the screening, we will calculate the risk of each patient according to variables already included in the standard pregnancy control. As there is no economic cost involved, we ensure universal accessibility for all pregnant women to screening for PE. However, the estimation of angiogenic markers is the strategy with the best performance when it comes to screening the risk of PE (21, 22). By adding PlGF determination (and blinded sFlT-1) to only a reduced proportion of patients, we believe that we can offer an early PE screening with maximum quality, while marginally increasing the economic cost. This design has been previously proposed after retrospective analysis of randomized clinical trials based on the universal PE screening with PlGF (25, 26). Our objective is to validate these results in a multicenter, real-world clinical setting study. This sample size assures enough statistical power for the main outcomes. We consider that the design and methodology of the project guarantees the external validity of the results.

Some limitations of our study need to be mentioned. First, our design is not a randomized control trial, therefore it does not include a control group without intervention. Several randomized clinical trials have already shown the efficacy of the first-trimester PE screening for the early implementation of low-dose ASA prophylaxis. Our objective is to validate a two-step screening system previously proposed by retrospectively analyzing high methodological quality clinical trials in a real clinical scenario. We have not considered the creation of a non-intervention group in the design, as our intention is to compare our results with all the screening strategies published to date. However, we have quality information on historical patient cohorts prior to the implementation of PE screening in all centers. As a contingency plan, if we consider it necessary at the end of the study, we could also compare the results with the group of patients in whom we have not been able to screen for PE in the first trimester for other reasons. We assume that the study design allows us less control over the results than a clinical trial, as it is subjected to the potential biases of routine clinical practice. However, we consider that this is, on the other hand, one of the differentiating points of this project, since it offers us results with high external validity and transferability to clinical reality. Second, for clinical purposes to be able to publish our results as soon as possible, follow-up of the offspring is limited to the neonatal period. However, this is not an important limitation, since it is not the objective of the project, and since all the study patients are registered, a long-term cohort study with these neonates could be considered.

## Ethics Statement

The studies involving human participants were reviewed and approved by Aragon Research Ethics Committee (CEICA) on 3 July 2020 (15/2020). The patients/participants provided their written informed consent to participate in this study.

## Author Contributions

EL, CT, MF, CL, and DO: conception and design of the research. DO: principal investigator. CT: co-investigator. SD, LR, AR-R, JM, TB, MF, AM, and SR-M: collaborators. CT, CL, and DO: wrote the first version of the manuscript, drafting, and revision of the manuscript. All authors revised it and contributed significantly in writing the final version that was accepted.

## Funding

This study was supported by the government of Spain, Plan Estatal de I+D+I and Instituto de Salud Carlos III- Subdirección General de Evaluación y Fomento de la Investigación Sanitaria, project PI19/00692, and the European Regional Development Fund (FEDER).

## Conflict of Interest

The authors declare that the research was conducted in the absence of any commercial or financial relationships that could be construed as a potential conflict of interest.

## Publisher's Note

All claims expressed in this article are solely those of the authors and do not necessarily represent those of their affiliated organizations, or those of the publisher, the editors and the reviewers. Any product that may be evaluated in this article, or claim that may be made by its manufacturer, is not guaranteed or endorsed by the publisher.
